# Supplementary material for: Arabidopsis ACINUS is O-glycosylated and regulates transcription and alternative splicing of regulators of reproductive transitions
Source: Nat Commun. 2021 Feb 11;12:945. doi: 10.1038/s41467-021-20929-7 (PMC7878923; doi:10.1038/s41467-021-20929-7)
Supplement: Supplementary file 1 — Supplementary Information [file 41467_2021_20929_MOESM1_ESM.pdf]

a Alignment of Acinus and AtACINUS, around SAP domain

Score	Expect	Method	Identities	Positives	Gaps
34.3 bits(77)	3e-05	Compositional matrix adjust.	18/41(44%)	25/41(60%)	0/41(0%)

```
Acinus:   66    TLDGKPLQALRVTDLKAALEQRGLAKSGQKSALVKRLKGAL  106
                 LD +P+   +VT+LK  L++R L   G K  LV+RL  AL
AtACINUS: 8     VLDNRPIDKWKVTELKEELKRRRLTTRGLKEELVRRLDEAL  48
```

Alignment of Acinus and AtACINUS, around RRM domain

Score	Expect	Method	Identities	Positives	Gaps
90.1 bits(222)	2e-22	Compositional matrix adjust.	35/82(43%)	58/82(70%)	2/82(2%)

```
Acinus:   1011  SNIVHISNLVRPFTLGQLKELLGRTGTLVEEAFWIDKIKSHCFVTYSTVEEAVATRTALH  1070
                +N + I   +RPFTL  ++ELLG+TG +   +FW+D IK+HC+V+Y +VEEA ATR A++
AtACINUS: 456   TNSLRIDRFLRPFTLKAVQELLGKTGNVT--SFWMDHIKTHCYVSYPSVEEAAATREAVY  513

Acinus:   1071  GVKWPQSNPKFLCADYAEQDEL  1092
                 ++WP +  + L A++   +E+
AtACINUS: 514   NLQWPPNGGRHLIAEFVRAEEV  535
```

Alignment of Acinus and AtACINUS, around RSB domain

Score	Expect	Method	Identities	Positives	Gaps
44.3 bits(103)	3e-08	Compositional matrix adjust.	18/29(62%)	23/29(79%)	0/29(0%)

```
Acinus:   1211  LDDLFRKTKAAPCIYWLPLTDSQIVQKEA  1239
                LDDLF+KTKA P IY+LPL++ Q+  K A
AtACINUS: 600   LDDLFKKTKAIPRIYYLPLSEEQVAAKLA  628
```

b Alignment of Pinin and AtPININ, around RSB domain

Score	Expect	Method	Identities	Positives	Gaps
67.4 bits(163)	3e-16	Compositional matrix adjust.	45/122(37%)	69/122(56%)	10/122(8%)

```
Pinin:   132  QNMDEKGKQRNRRIFGLLMGTLQKFKQEST--VATERQKRRQEIEQKLEVQAEEERKQVE  189
              +N D K   RNRR+ G L+GTL+KF++E      T+   RR    Q+ E +A EE +++
AtPININ: 153  KNEDPKLVNRNRRMLGNLLGTLEKFRKEDKQRSGTDAYARRTAALQRAEEKAREESERLR  212

Pinin:   190  NERRELFEERRAKQTELRLL------EQKVELAQLQEEWNEHNAKIIKYIRTKTKPHLFY  243
               + RE   E+R +   LR        ++K+EL  LQ  W+EH  K+  +IRTK +P ++Y
AtPININ: 213  LQERENLTEKRRRDLTLRARVAAKAEQKKLELLFLQ--WSEHQKKLSNFIRTKAEPRIYY  270

Pinin:   244  IP  245
               P
AtPININ: 271  AP  272
```

c

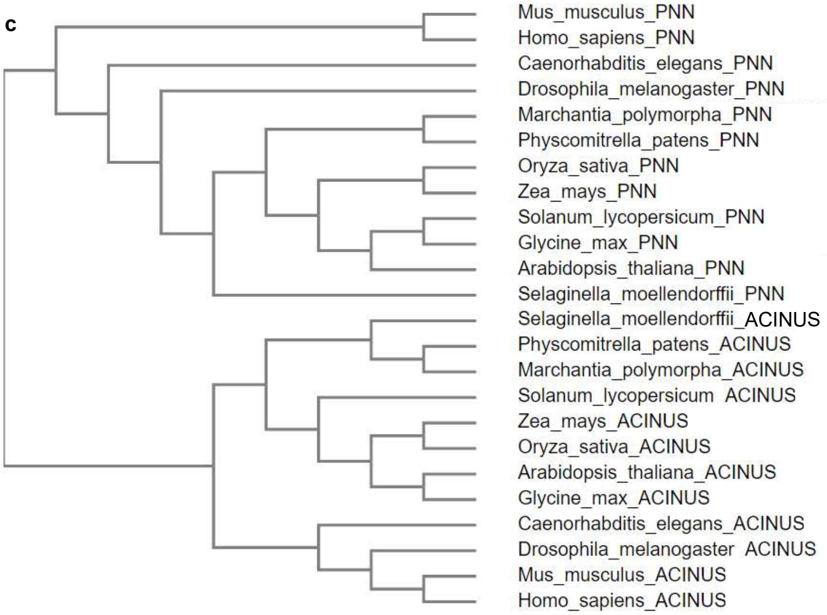

Supplementary Fig. 1 | Protein sequence analysis of AtACINUS and AtPININ. (a,b) Pairwise sequence alignment between human Acinus and AtACINUS and between human Pinin and AtPININ using Blastp from NCBI blastp suite. Hits with E value<0.01 are shown. **(c)** Dendrogram of AtACINUS and AtPININ homologs from various species. PNN=PININ.

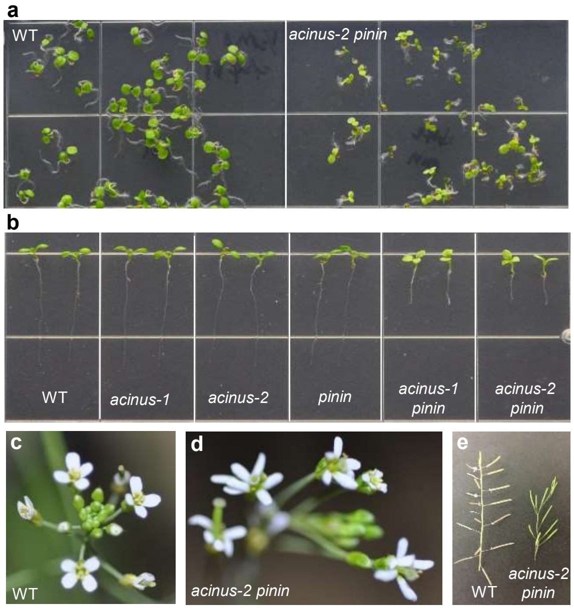

Supplementary Fig. 2 | Pleiotropic developmental defects in the *acinus-2 pinin-1* mutant. (a) Germination of *acinus-2 pinin-1* seeds was slightly delayed compared to WT. **(b)** The *acinus-2 pinin-1* mutants showed short root and tri-cotyledon phenotypes. **(c,d)** The *acinus-2 pinin-1* double mutant (d) showed increased number of petals compared to WT (c). **(e)** The *acinus-2 pinin-1* double mutant (right) showed phyllotaxis defects compared to WT (left).

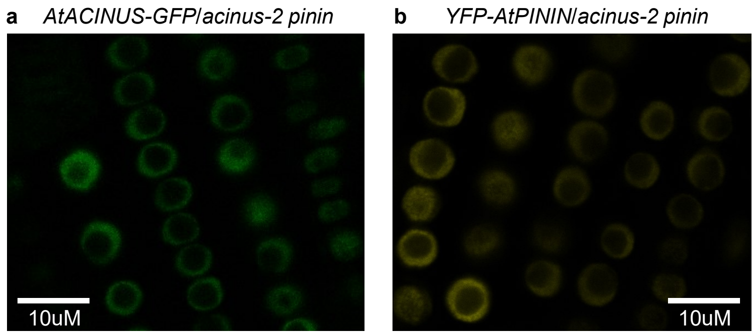

Supplementary Fig. 3 | Confocal image of AtACINUS-GFP localization in the root of *AtACINUS-GFP/acinus-2 pinin-1* seedlings (a) and YFP-PININ localization in the root of *YFP-PININ/acinus-2 pinin-1* seedlings (b).

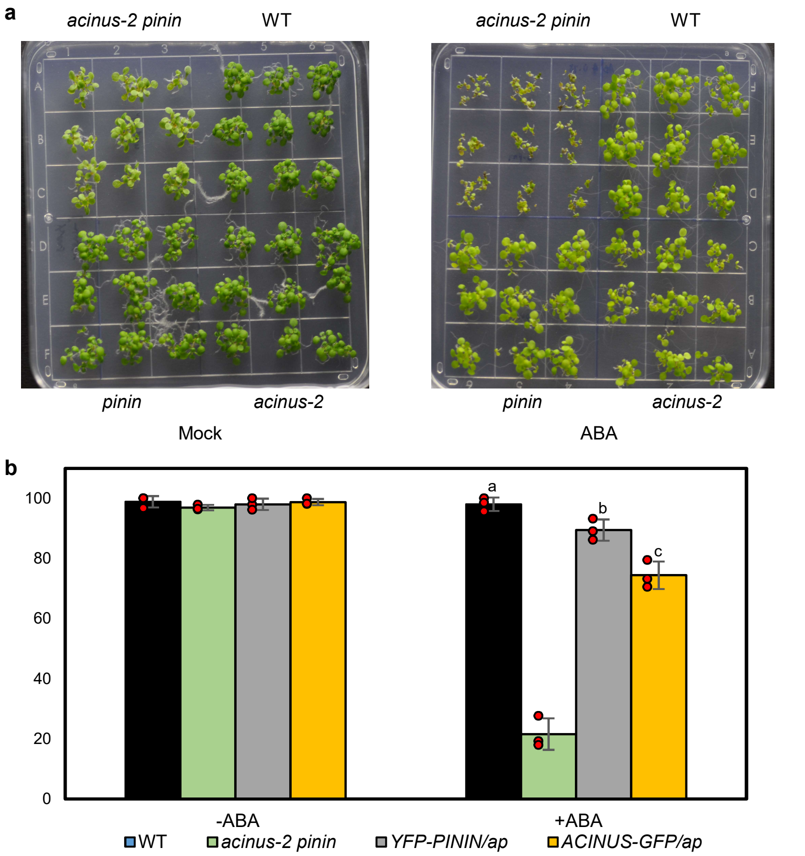

Supplementary Fig. 4 | Post-germination seedling growth is inhibited by ABA in *acinus-2 pinin-1.* (a) Seeds of WT, *acinus-2*, *pinin-1* and *acinus-2 pinin-1* were germinated on filtered paper, transferred to mock medium or medium containing 1µmol/L ABA and grown for 5 days. **(b)** Germination rate of the indicated genotypes on ½ MS medium containing 0 or 0.5 µmol/L ABA for six days. Values represent Mean±SD calculated from 3 biological replicates (n=3). Statistically significant differences to *acinus-2 pnn-1* were determined by two-tailed *t* test. The *P* values for a, b and c in are 3.55E-4, 1.28E-4 and 2.18E-4.

a

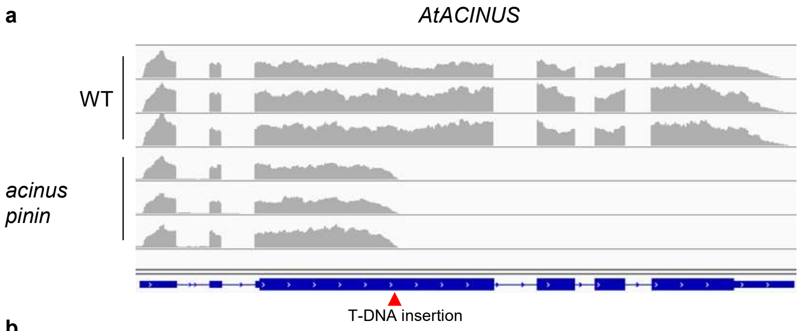

b

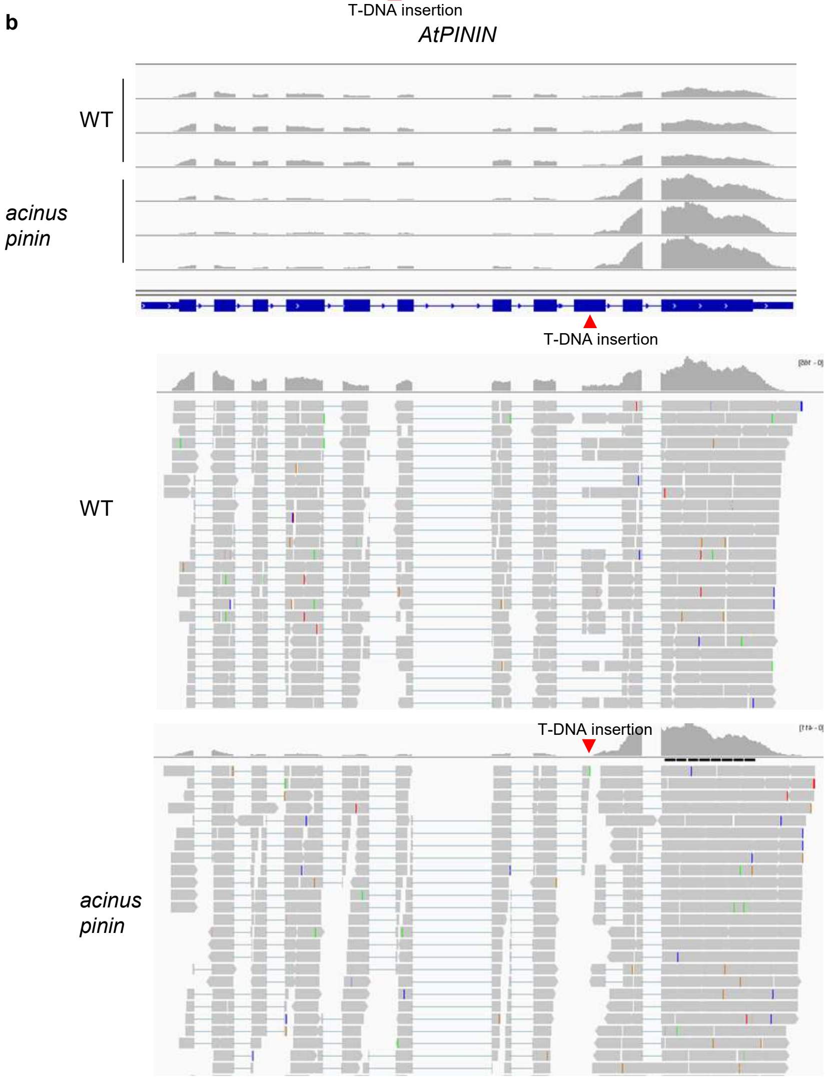

Supplementary Fig. 5 | Full length *AtACINUS* and *AtPININ* were not transcribed in *acinus-2 pinin-1*. (a) A partial *AtACINUS* transcript from the 5' transcription start site until T-DNA insertion site was detected in *acinus-2 pinin-1*. **(b)** A partial *AtPININ* transcript from the 5' transcription start site until T-DNA insertion site was detected at a reduced level in *acinus-2 pinin-1*. Transcription was initiated from the T-DNA insertion to transcribe the 3' end of *AtPININ* after the T-DNA insertion site at an increased level. However, there was no full length *AtPININ* produced because transcripts were discontinuous and showed a gap in the 9th exon at the position marked by the red triangle. No reads spanning (gray bar or blue line) this region was detected in *acinus-2 pinin-1* while a large number of reads spanning this region were detected in WT.

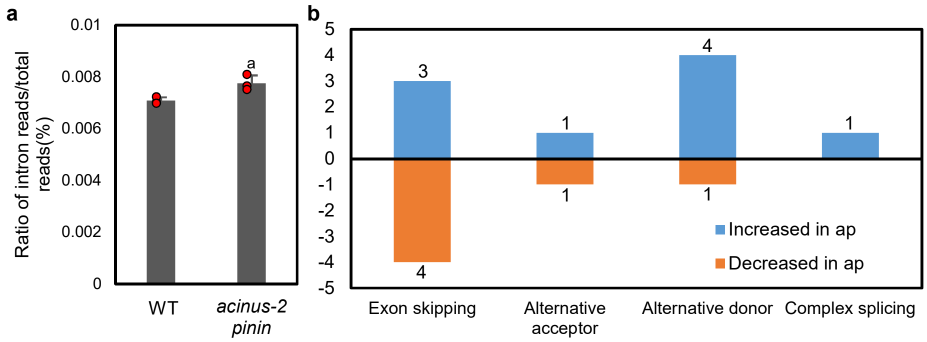

Supplementary Fig. 6 (**a**) The percentage of intron reads in WT and the *acinus-2 pinin-1* double mutant. Values represent Mean±SD calculated from 3 biological replicates (n=3). Statistically significant difference to WT was determined by two-tailed *t* test. The *P* values for a is 4.56E-2. (**b**) A summary of other types of splicing defects in *acinus-2 pinin-1* compared to WT.

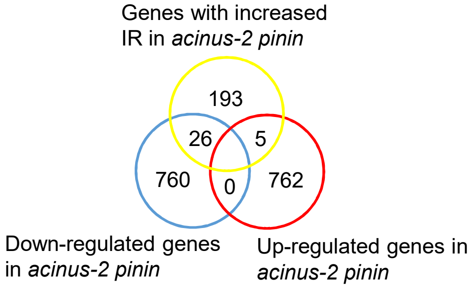

Supplementary Fig. 7 | Overlap between differentially expressed genes in *acinus-2 pinin-1* and genes with increased intron retention in *acinus-2 pinin-1*.

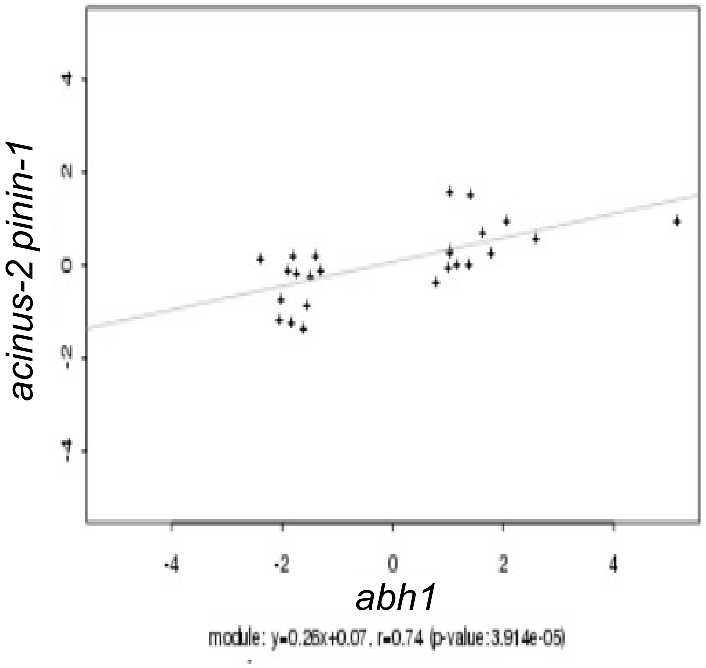

Supplementary Fig. 8 | Genes mis-regulated in *abh1* shows a strong correlation to genes mis-regulated in *acinus-2 pinin-1*, with Spearman's correlation=0.74.

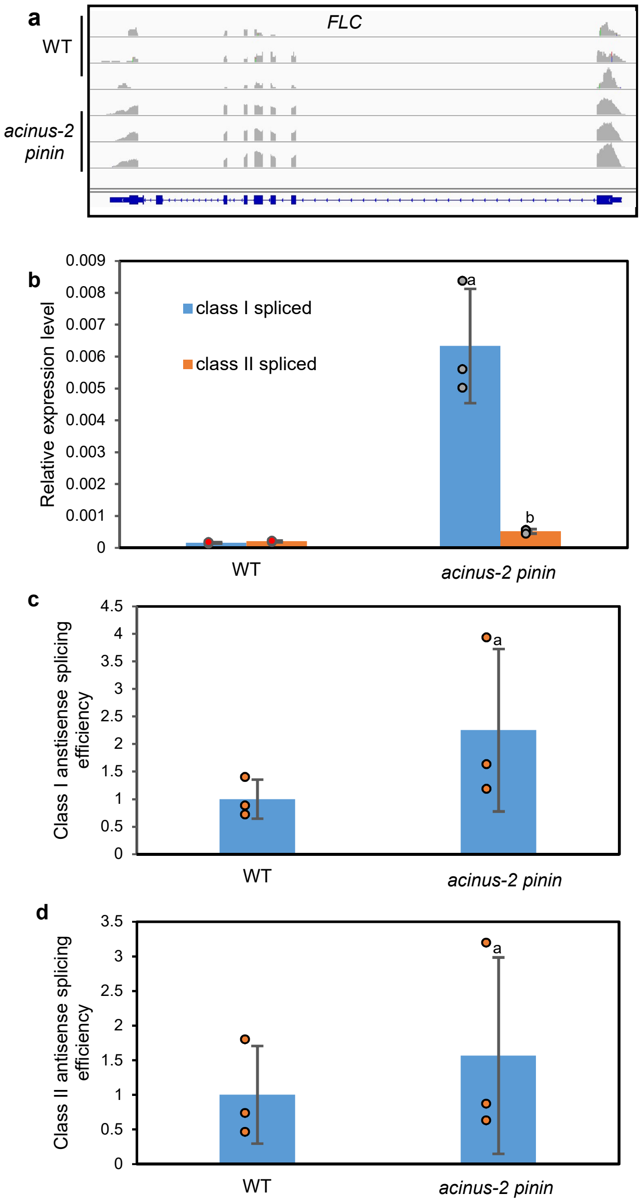

Supplementary Fig. 9 | *FLC* antisense I is increased relative to antisense II in *acinus-2 pinin-1* while the splicing efficiency is not significantly changed. (a) Reads coverage of *FLC* locus in WT and *acinus-2 pinin-1*. Track height is set to 15 in WT and 200 in *acinus-2 pinin-1*. **(b)** Expression levels of *FLC* spliced class I antisense and spliced class II antisense relative to *PP2a* in WT and *acinus-2 pinin-1*. Statistically significant differences to WT were determined by two-tailed *t* test. The *P* values for a and b in are 2.70E-2 and 8.60E-3. **(c)** Class I antisense splicing efficiency calculated from class I spliced/class I unspliced. WT is set to 1. Statistically significant difference to WT was determined by two-tailed *t* test. The *P* values for a is 0.28. **(d)** Class II antisense splicing efficiency calculated from class II spliced/class II unspliced. WT is set to 1. In our experimental conditions, only class II-II is detected and used for calculation for class II antisense. Statistically significant difference to WT was determined by two-tailed *t* test. The *P* values for a is 0.58. RNA was extracted from 12-day-old seedlings. Values represent Mean±SD calculated from 3 biological replicates (n=3) for (b-d).

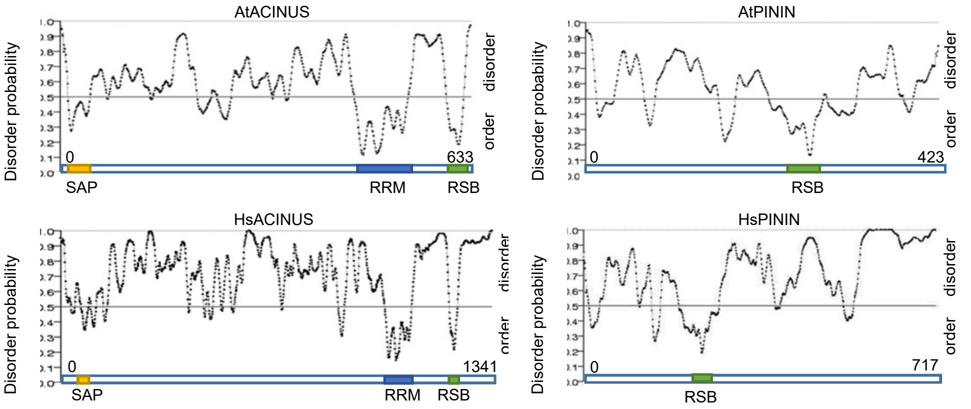

Supplementary Fig. 10 | ACINUS and PININ are predicted to be highly disordered proteins with small ordered regions that overlap with functional domains.

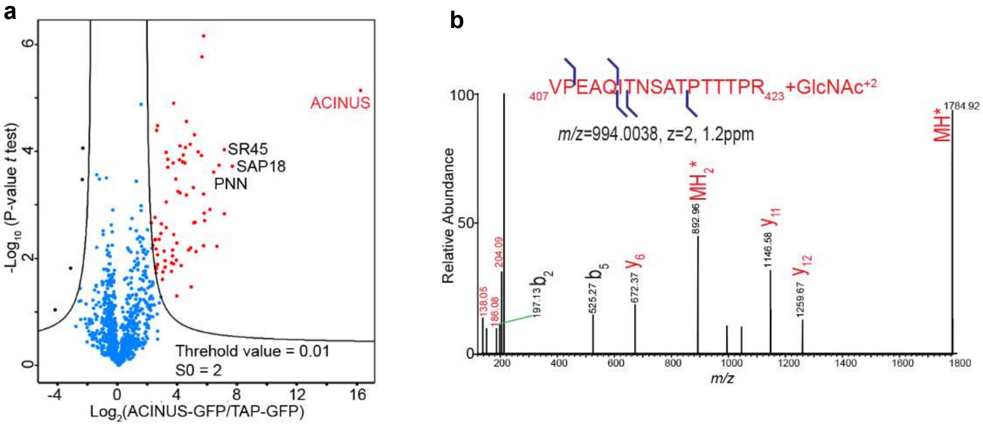

Supplementary Fig. 11 | (a) Volcano plot of the IP-MS analysis of the AtACINUS interactome. The logarithmic ratios of protein signal intensities between AtACINUS-GFP and TAP-GFP (negative control) are plotted against negative logarithmic p-values of the *t*-test of triplicate IP-MS. The hyperbolic curves are based on an FDR estimation 0.01 and S0=2. The curves separate bait AtACINUS and its specific interactors (red dots) from background proteins (blue dots) and possible false positive (black dots) that are enriched in the TAP-GFP control. Additional information is in Supplemental Data 1. **(b)** HCD spectra detected O-GlcNAcylation on a sequence spanning amino acid 407 to 423 of AtACINUS with neutral loss.

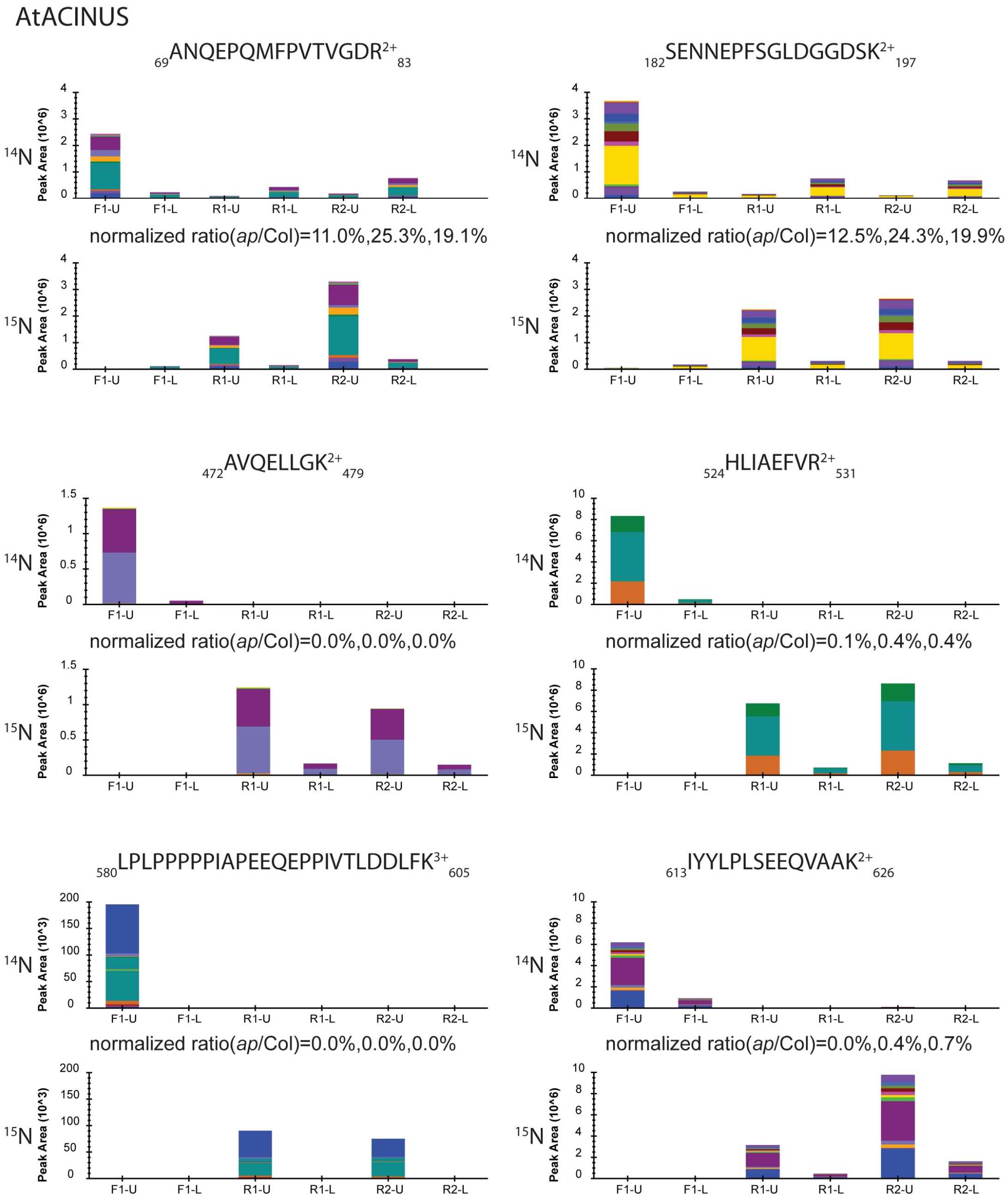

Supplementary Fig.12| Targeted quantifications using Parallel Reaction Monitoring (PRM) show AtACINUS N-terminal has reduced expression and C-terminal is undetectable in *acinus-2 pinin-1* mutant. Two gel segments (upper part (U) and lower part(L)) were excised from each mixed samples and subjected to trypsin digestion. Proteins were quantified from both segments of each mixed sample, including F1 (^{14}N Col/ ^{15}N *acinus-2 pinin-1*) and R1, R2 samples (^{14}N *acinus-2 pinin-1*/ ^{15}N Col). Peak areas of fragments were extracted for the ^{14}N and ^{15}N labeled peptides of targeted proteins using 5 ppm mass window and integrated across the elute profile using Skyline platform. The sum of peak areas from two segments were calculated from Col and *acinus-2 pinin-1* peptides and ratios were calculated and normalized to Tubulin.

AtPININ

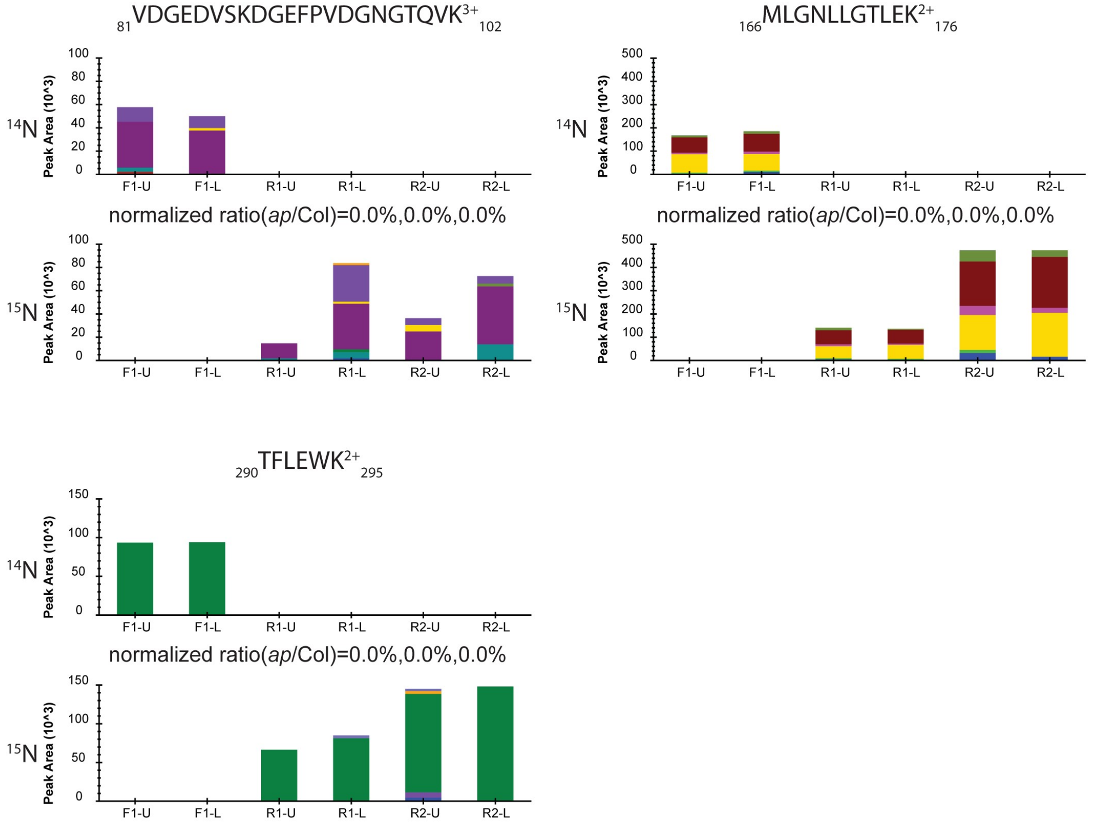

Control Tubulin 2

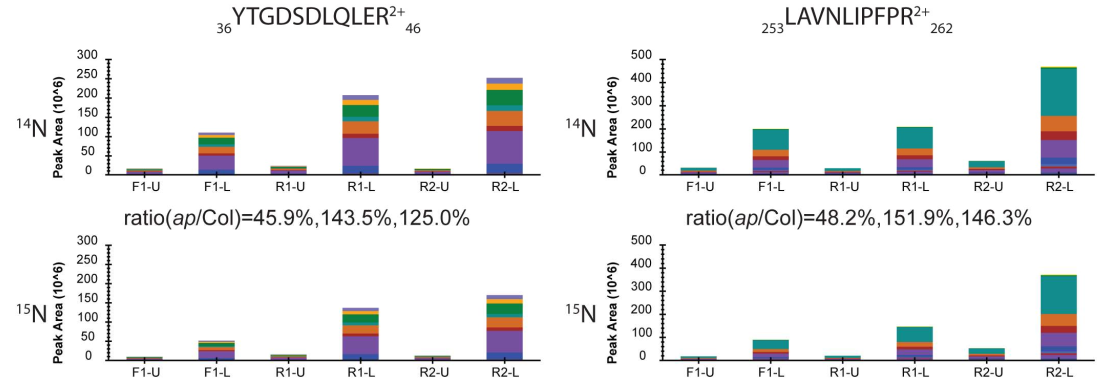

Supplementary Fig.13| Targeted quantifications using Parallel Reaction Monitoring (PRM) show AtPININ protein level is non-detectable in *acinus-2 pinin-1* mutant. Two gel segments (upper part (U) and lower part(L)) were excised from each mixed samples and subjected to trypsin digestion. Proteins were quantified from both segments of each mixed sample, including F1 (^{14}N Col/ ^{15}N *acinus-2 pinin-1*) and R1, R2 samples (^{14}N *acinus-2 pinin-1*/ ^{15}N Col). Peak areas of fragments were extracted for the ^{14}N and ^{15}N labeled peptides of targeted proteins using 5 ppm mass window and integrated across the elute profile using Skyline platform. The sum of peak areas from two segments were calculated from Col and *acinus-2 pinin-1* peptides and ratios were calculated and normalized to Tubulin.

SR45

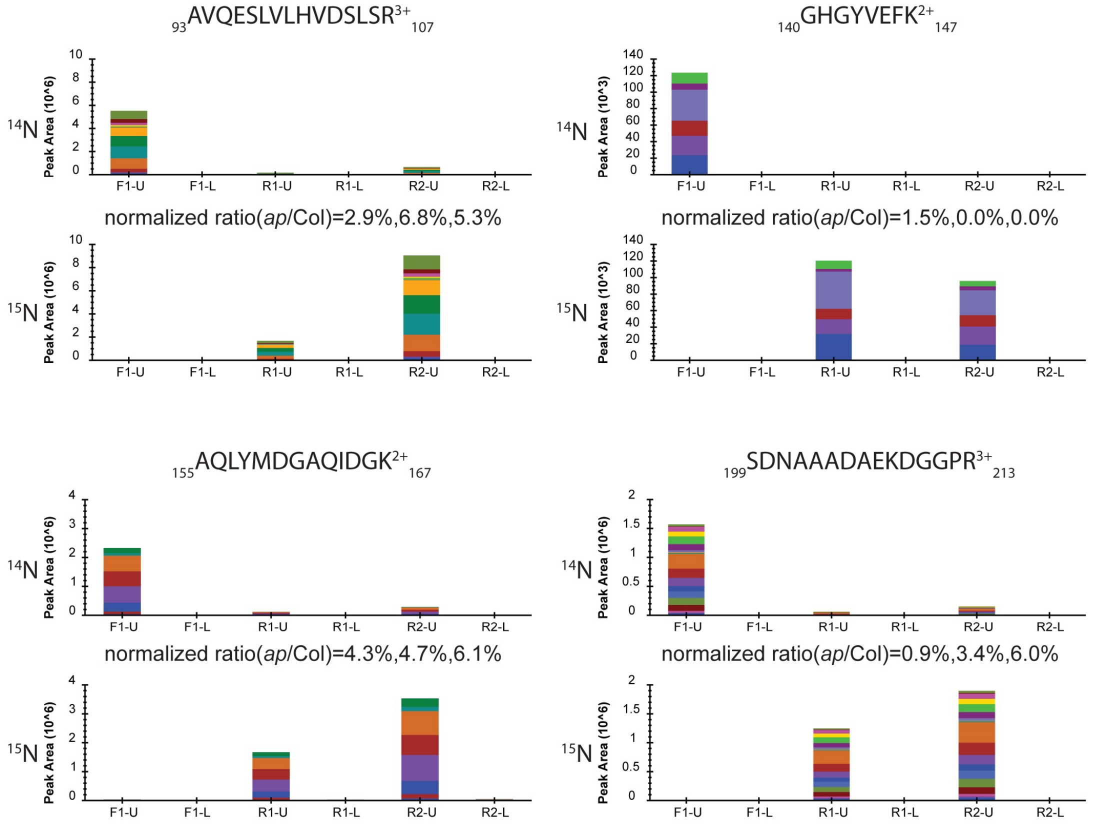

Supplementary Fig.14| Targeted quantifications using Parallel Reaction Monitoring (PRM) show much reduced SR45 protein levels in *acinus-2 pinin-1* mutant. Two gel segments (upper part (U) and lower part(L)) were excised from each mixed samples and subjected to trypsin digestion. Proteins were quantified from both segments of each mixed sample, including F1 (^{14}N Col/ ^{15}N *acinus-2 pinin-1*) and R1, R2 samples (^{14}N *acinus-2 pinin-1*/ ^{15}N Col). Peak areas of fragments were extracted for the ^{14}N and ^{15}N labeled peptides of targeted proteins using 5 ppm mass window and integrated across the elute profile using Skyline platform. The sum of peak areas from two segments were calculated from Col and *acinus-2 pinin-1* peptides and ratios were calculated and normalized to Tubulin.

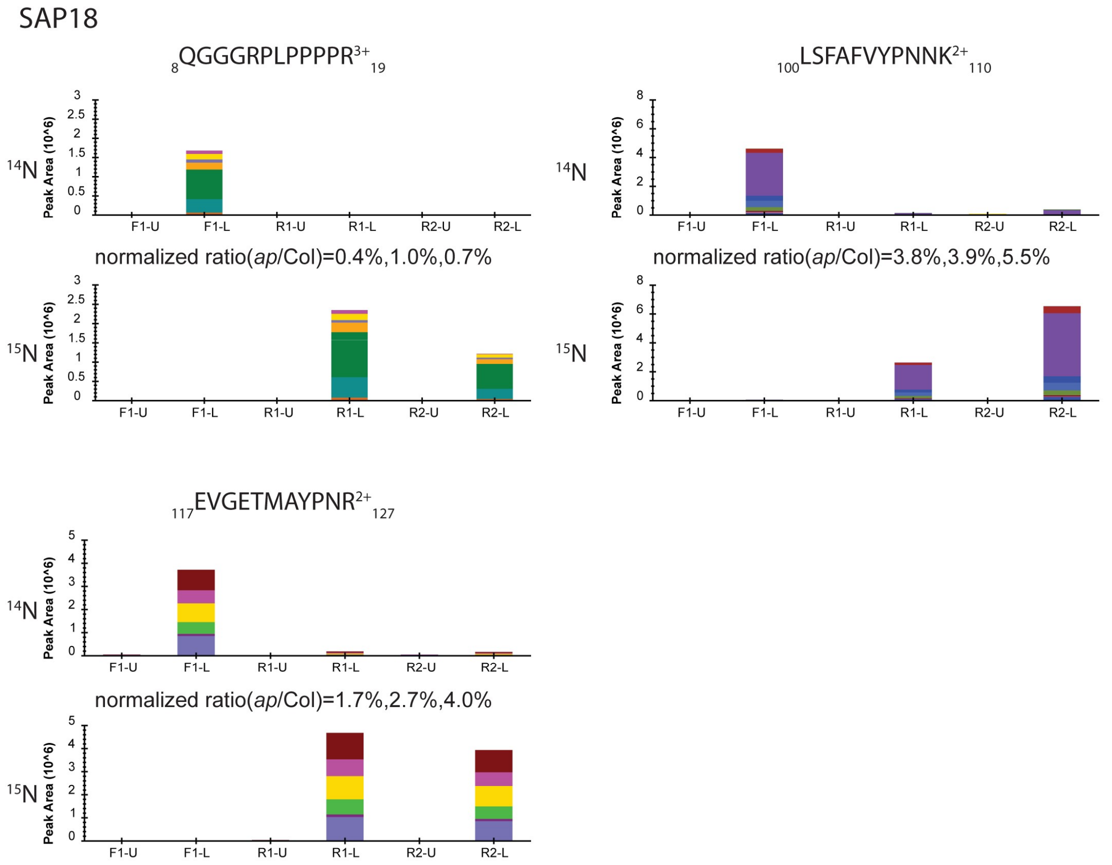

Supplementary Fig.15| Targeted quantifications using Parallel Reaction Monitoring (PRM) show much reduced SAP18 protein levels in *acinus-2 pinin-1* mutant. Two gel segments (upper part (U) and lower part(L)) were excised from each mixed samples and subjected to trypsin digestion. Proteins were quantified from both segments of each mixed sample, including F1 (^{14}N Col/ ^{15}N *acinus-2 pinin-1*) and R1, R2 samples (^{14}N *acinus-2 pinin-1*/ ^{15}N Col). Peak areas of fragments were extracted for the ^{14}N and ^{15}N labeled peptides of targeted proteins using 5 ppm mass window and integrated across the elute profile using Skyline platform. The sum of peak areas from two segments were calculated from Col and *acinus-2 pinin-1* peptides and ratios were calculated and normalized to Tubulin.

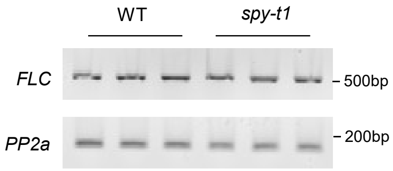

Supplementary Fig.16 Semi-quantitative RT-PCR of *FLC* in WT and *spy-t1*. *PP2a* serves as an internal control.
