# Supplementary material for: Arabidopsis ACINUS is O-glycosylated and regulates transcription and alternative splicing of regulators of reproductive transitions
Source: Nat Commun. 2021 Feb 11;12:945. doi: 10.1038/s41467-021-20929-7 (PMC7878923; doi:10.1038/s41467-021-20929-7)
Supplement: Supplementary file 3 — Descriptions of Additional Supplementary Files [file 41467_2021_20929_MOESM3_ESM.pdf]

Descriptions of Additional Supplementary Files

Supplementary data 1

Description: Summary of reads mapping quality and differentially expressed genes in acinus-2 pinin-1 identified by RNA-seq analysis.

Supplementary data 2

Description: Alternative splicing events in acinus-2 pinin-1 identified by RNA-seq analysis**.**

Supplementary data 3

Description: AtACINUS interactome.

Supplementary data 4

Description: List of primers used in the study.

Supplementary data 5

Description: Targeted quantification on AtACINUS, AtPININ, SR45 and AtSAP18 between Col and acinus-2 pinin-1 mutant.
